# Reversible Trifokalität durch das Duett-Verfahren

**DOI:** 10.1007/s00347-020-01096-4

**Published:** 2020-04-15

**Authors:** Ramin Khoramnia, Timur M. Yildirim, Hyeck-Soo Son, Grzegorz Łabuz, Christian S. Mayer, Gerd U. Auffarth

**Affiliations:** International Vision Correction Research Centre (IVCRC), Universitäts-Augenklinik Heidelberg, Im Neuenheimer Feld 400, 69120 Heidelberg, Deutschland

**Keywords:** Trifokale IOL, IOL Tausch, Presbyopie, Sulcoflex, Additive IOL, Trifocal IOL, IOL exchange, Presbyopia, Sulcoflex, Supplementary IOL

## Abstract

Funktionelle Ergebnisse und das Auftreten von Nebenwirkungen (insbesondere photischer Phänomene) können beim Einsatz multifokaler Intraokularlinsen (IOL) schwer vorhergesagt werden. Des Weiteren können Patienten im Laufe des Lebens Erkrankungen entwickeln, bei denen eine multifokale Optik von Nachteil sein kann. In diesen Fällen ist ein Austausch der IOL die einzige Therapieoption. Die Implantation einer monofokalen oder monofokal-torischen IOL in den Kapselsack und einer additiven trifokalen IOL in den Sulcus ciliaris in einer als Duett-Verfahren bezeichneten Operation ermöglicht Trifokalität, die bei Bedarf vergleichsweise einfach wieder rückgängig gemacht werden kann.

**Video online:**

Die Online-Version dieses Beitrags (10.1007/s00347-020-01096-4) enthält ein Video.

## Abstract (en)

The functional results and the occurrence of side effects (especially photic phenomena) for multifocal intraocular lenses (IOL) are difficult to predict. Furthermore, in the course of life patients can develop diseases in which a multifocal optic would be a disadvantage. In these cases exchange of the IOL is the only treatment option. Implantation of a monofocal or monofocal toric IOL in the capsular bag and a supplementary trifocal IOL in the ciliary sulcus in a single operation, known as a duet procedure, provides trifocality that can be easily reversed if necessary.

## Trifokale Optiken

Die Implantation multifokaler Intraokularlinsen (IOL) in den Kapselsack kann Patienten Brillenunabhängigkeit in mehreren Distanzen ermöglichen [13].

 Unter den multifokalen IOL haben die diffraktiven Trifokallinsen diffraktive Bifokallinsen zum Erreichen einer Brillenunabhängigkeit weitgehend abgelöst, da sie sowohl in klinischen als auch in Laboruntersuchungen bessere Ergebnisse (v. a. im Intermediärbereich), aber keine zusätzlichen Nachteile zeigten [3, 5, 10]. Der Anteil an implantierten kapselsackfixierten Multifokallinsen in Deutschland liegt in den letzten Jahren relativ konstant in einem Bereich von 3 % [17]. Neben wirtschaftlichen Gründen für die relativ niedrigen Zahlen ist auch die Tatsache dafür verantwortlich, dass nicht jeder Patient für eine multifokale Optik geeignet ist. Zu bekannten Nachteilen multifokaler Optiken zählen das vermehrte Auftreten photischer Phänomene sowie eine im Vergleich zu monofokalen Linsen geringere Toleranz gegenüber nichtoptimalen Bedingungen, wie beispielsweise einer Dezentrierung [1, 15].

## Notwendigkeit einer Reversibilität

Im Falle des Auftretens von Nebenwirkungen, welche auf das optische Prinzip zurückzuführen sind, ist eine Explantation der Linse aus dem Kapselsack bisher die einzige Therapieoption. Dies ist jedoch mit einer erhöhten Komplikationsrate verbunden [4]. Besonders nachdem die Linse im Kapselsack eingewachsen ist, kann ein IOL-Austausch erschwert sein.

Außerdem können Patienten im Laufe des Lebens Erkrankungen entwickeln, bei denen eine multifokale Optik von Nachteil sein kann. Auch in diesen Fällen wäre eine einfachere Reversibilität wünschenswert.

Aus diesen Gründen werden augengesunden Patienten diffraktive trifokale IOL von den meisten Chirurgen erst nach eingehender Aufklärung und bei explizitem Wunsch nach Brillenunabhängigkeit angeboten.

## Einsatzgebiete additiver IOL

Nach einer Kataraktoperation mit Implantation einer kapselsackfixierten Intraokularlinse (IOL) können zusätzlich spezielle additive Linsen vor die primäre IOL in den Sulcus ciliaris implantiert werden. Für diese additiven sulcusgestützten IOL existieren verschiedene Einsatzgebiete:

1. Brechkraftfehler pseudophaker Augen können korrigiert werden [8].
2. Multifokale Optiken können neben einem Brechkraftfehler auch die Presbyopie pseudophaker Patienten behandeln [7]. Bisherige Linsenmodelle konnten dafür durch refraktive und/oder diffraktive Optiken nur 2 Foci (Fern- und Nahbereich) generieren.
3. In spezieller Ausführung mit sehr hoher Nahaddition (Scharioth-IOL) können die additiven IOL außerdem zur visuellen Rehabilitation bei der altersbedingten Makuladegeneration (AMD) eingesetzt werden [12].
4. Weiterhin können die additiven sulcusgestützten IOL in einem sog. Duett-Verfahren zur Herstellung einer reversiblen Multifokalität verwendet werden [2, 6, 11, 19]. Dieses Verfahren unter Verwendung einer trifokalen additiven IOL soll im Verlauf näher erläutert werden.

## Das Duett-Verfahren

**Figure 1:**
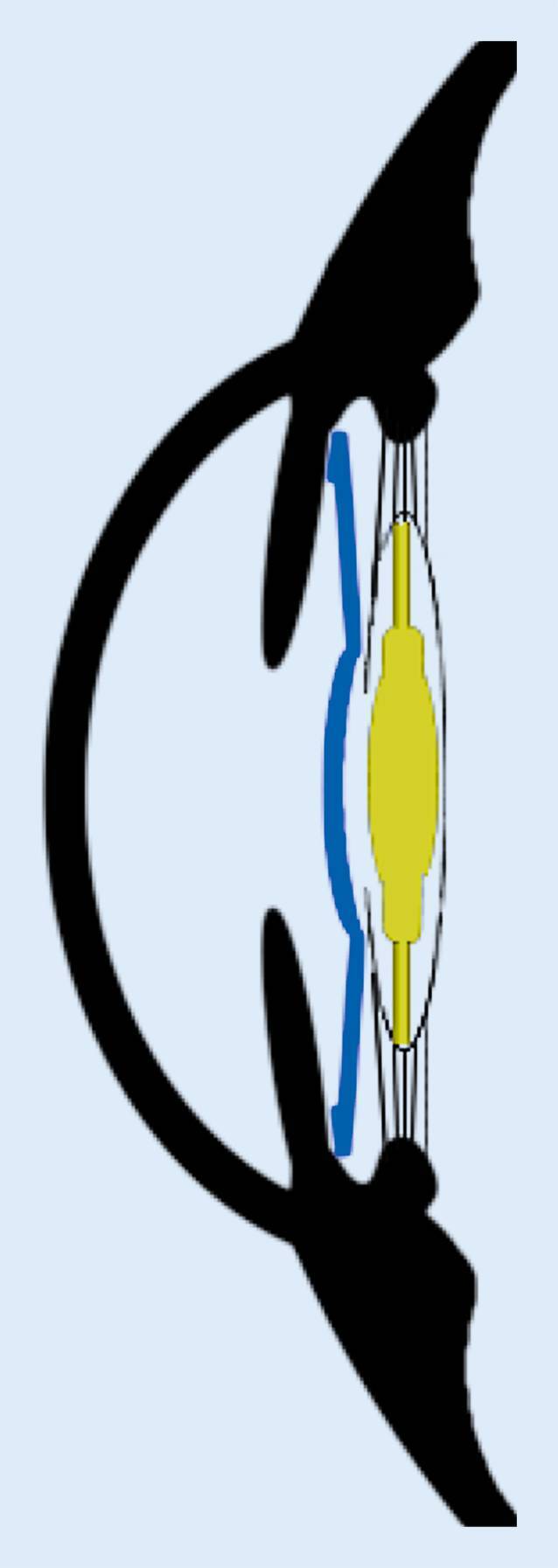

Die Implantation einer monofokalen oder monofokal-torischen IOL in den Kapselsack und einer additiven multifokalen IOL in den Sulcus ciliaris in einer Sitzung bietet im Rahmen eines sog. Duett-Verfahrens die Möglichkeit, Patienten mit einer multifokalen Optik zu versorgen, die bei Bedarf einfacher beseitigt werden kann. Dafür wird nach Entfernung der körpereigenen Linse eine primäre IOL (monofokal oder torisch) in den Kapselsack implantiert. Anschließend erfolgt das Einsetzen einer speziell für den Sulcus ciliaris entwickelten additiven IOL vor die primäre kapselsackfixierte IOL (Abb. 1). Je nachdem, in welcher Distanz die beste unkorrigierte Sehschärfe im Falle einer Explantation der additiven Linse liegen soll, können die Stärken der beiden IOL unterschiedlich kombiniert werden. Soll die Ferne optimal korrigiert sein, wird die Zielrefraktion der monofokalen/monofokal-torischen Kapselsack-IOL auf Emmetropie gezielt, und als multifokale additive Linse wird eine IOL ohne zusätzliche Basisbrechkraft gewählt. In manchen Fällen kann es sinnvoll sein, die Zielrefraktion der Kapselsack-IOL in einem myopen Bereich zu wählen, um im Falle einer Explantation der additiven Linse den unkorrigierten Nahvisus zu optimieren. Dann muss die Basisbrechkraft der additiven IOL so gewählt werden, dass mit beiden Linsen zusammen Emmetropie erreicht wird. Dieses Vorgehen ist z. B. bei (hoch) myopen Patienten sinnvoll, welche einen vergleichsweise guten Seheindruck in der Nähe gewohnt sind.

Bisher standen für das Duett-Verfahren ausschließlich bifokale Linsen verschiedener Hersteller zur Verfügung. Diffraktive bifokale Optiken sind funktionell den trifokalen, insbesondere aufgrund des fehlenden Intermediärfokus, unterlegen. Da Tätigkeiten im Intermediärbereich (z. B. PC-Arbeit, der Einsatz von Smartphones und Tablets etc.) in den letzten Jahren zunehmend an Bedeutung gewonnen haben, wünschen Patienten gerade in diesem Abstand zunehmend Brillenunabhängigkeit.

## Sulcoflex trifocal

Seit Kurzem ist nun auch die weltweit erste trifokale additive diffraktive Intraokularlinse für die Implantation in den Sulcus ciliaris verfügbar. Der Aufbau der Linse entspricht dem der vorangegangenen Sulcoflex IOL-Modelle (Rayner, Hove, UK), welche speziell für die Implantation in den Sulcus ciliaris bei pseudophaken Augen entworfen wurden. In die Optik der Linse wurden diffraktive Ringe eingearbeitet, welche neben dem Fernfokus 2 zusätzliche Brennpunkte generieren. Die Spezifikationen des trifokalen Modells der IOL (Sulcoflex trifocal, Rayner, Hove, UK) sind in Tab. 1 zusammengefasst. Mit dieser Linse kann nun erstmals ein Verfahren angeboten werden, welches eine reversible Trifokalität ermöglicht. Im Folgenden soll das operative Vorgehen des Duett-Verfahrens unter Verwendung der neuen Sulcoflex trifocal vorgestellt werden.

**Table 1:** 

*Aufbau*	Einstückig, 14,0 mm Gesamtdurchmesser
*Haptikkonfiguration*	10° posteriore Angulation
*Verfügbare Linsenstärken*	−3,0 dpt bis +3,0 dpt (in 0,5-dpt-Schritten)
	−1,0 dpt bis +1,0 dpt (in 0,25-dpt-Schritten)
*Material*	Hydrophiles Acrylat (26 % Wassergehalt)
*Optik*	
Durchmesser	6,5 mm
Form	Anterior konvex, posterior konkav
Multifokale Technologie	Zentrale 4,5-mm-Zone mit 16 diffraktiven Ringen
Additionen	+3,5 dpt und +1,75 dpt auf IOL-Ebene
Asphärizität	Aberrationsneutral

## Operatives Vorgehen

**Figure 2:**
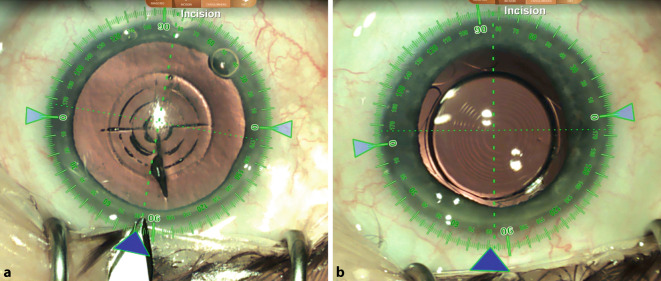

Wie im präsentierten Beispielfall können ein Femtosekundenlasersystem (LenSx Laser, Alcon, Fort Worth, TX, USA) und ein digitales Markierungssystem (Verion, Alcon, Fort Worth, TX, USA) eingesetzt werden, um das refraktive Ergebnis zu optimieren. Grundsätzlich kann die Technik aber auch ohne unterstützende Systeme Anwendung finden. Zunächst wird mit dem Femtosekundenlaser die Kapsulotomie (5 mm Durchmesser) angelegt sowie die Vorfragmentierung des Linsenkerns durchgeführt. Anschließend wird das Auge mit dem Führungssystem digital markiert, um den Hauptschnitt so zu platzieren, dass ein bestehender (geringer) kornealer Zylinder optimal korrigiert wird. Die wichtigsten der folgenden Schritte der Operation sind in Video 1 zusammengestellt: Durch das Führungssystem werden wichtige Informationen in das Operationsmikroskop eingeblendet, z. B. die ideale Position des Tunnelschnitts zur Optimierung des kornealen Astigmatismus. Im vorliegenden Fall wurde eine 2,5-mm-Clear-Cornea-Tunnel-Inzision bei 81° angelegt (Abb. 2a). Nach Entfernung der Linse werden der Kapselsack und die Vorderkammer mit einem (kohäsiven) Ophthalmic Viscoelastic Device (OVD) gestellt und eine monofokale IOL (hier: +19,0 dpt RayOne RA0800C [Rayner, Hove, UK]) in den Kapselsack implantiert. Anschließend erfolgt die Entfernung des OVD mit besonderem Augenmerk auf eine komplette Entfernung auch hinter der primären Linse. Dann wird der Sulcus ciliaris mit (kohäsivem) OVD gestellt und die additive Sulcoflex trifocal IOL, in diesem Fall ohne zusätzliche Basisbrechkraft, implantiert. Bei diesem Schritt muss darauf geachtet werden, dass die additive Linse in den Sulcus ciliaris und nicht versehentlich (teilweise) in den Kapselsack implantiert wird, da der entstehende Tilt oder die Dezentrierung zu einem schlechteren funktionellen Ergebnis führen würden. Anschließend wird das OVD – auch zwischen den beiden Linsen – wieder entfernt (Abb. 2b). Es erfolgt eine medikamentöse Pupillenverengung (mit Acetylcholin), um das Risiko eines postoperativen Optic Capture zu vermeiden.

## Fallbeispiel

### Präoperative Daten

Ein 18-jähriger Patient stellte sich mit beidseitiger Katarakt aufgrund eines Hyperferritinämie-Katarakt-Syndroms mit zunehmender Visusminderung und vermehrter Blendung in unserer Sprechstunde vor. Aufgrund des jungen Alters und des damit einhergehenden Akkommodationsverlusts durch eine Kataraktoperation besprachen wir die Möglichkeit des Einsatzes multifokaler Optiken. Nach sorgfältiger Abwägung entschieden wir uns zusammen mit dem Patienten für das oben beschriebene Duett-Verfahren mit Implantation torisch-monofokaler IOL in den Kapselsack und additiver diffraktiver trifokaler IOL in den Sulcus ciliaris an beiden Augen mit emmetroper Zielrefraktion für die Kapselsack-IOL.

### Postoperatives Ergebnis

**Figure 3:**
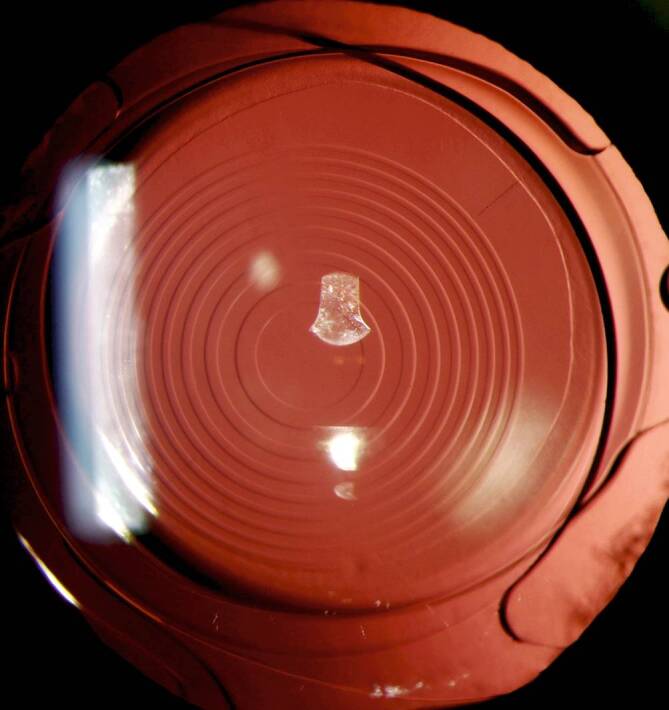

Drei Monate postoperativ wurden gute Ergebnisse für den unkorrigierten Fern‑, Intermediär- und Nahvisus erreicht mit minimaler verbliebener subjektiver Refraktion (Tab. 2). Der Patient war subjektiv sehr zufrieden, und auch die photischen Phänomene waren vergleichbar mit denen einer diffraktiven trifokalen kapselsackgestützten IOL. Die Abb. 3 zeigt das postoperative Spaltlampenbild des rechten Auges in Retroillumination.

**Table 2:** 

*Subjektive Refraktion*		
Rechtes Auge		Linkes Auge
−0,25/−0,25/110°		Plan/−0,25/80°
*Unkorrigierter Fernvisus (6* *m)*		
Rechtes Auge	Binokular	Linkes Auge
0,8	1,0	0,8
*Unkorrigierter Intermediärvisus (80* *cm)*		
Rechtes Auge	Binokular	Linkes Auge
1,0	1,0	1,0
*Unkorrigierter Nahvisus (40* *cm)*		
Rechtes Auge	Binokular	Linkes Auge
1,0	1,0	1,0

## Diskussion

Bei dem hier vorgestellten Duett-Verfahren werden in einer Sitzung eine monofokale oder monofokal-torische IOL in den Kapselsack und eine trifokale additive IOL in den Sulcus ciliaris implantiert. Der Vorteil dieses Vorgehens besteht in der Schaffung einer reversiblen Trifokalität, da die additive IOL auch Jahre nach dem Eingriff ohne größeren Aufwand entfernt werden könnte.

### Einsatzgebiete additiver IOL

Additive sulcusgestützte IOL werden seit Jahren eingesetzt, um das refraktive Ergebnis nach erfolgter primärer IOL-Implantation zu verbessern. Kahraman et al. setzten in einer Studie die monofokale asphärische Sulcoflex 653L ein und verbesserten so das mittlere sphärische Äquivalent in ihrem Kollektiv von −1,25 dpt auf −0,25 dpt [8]. Besonders nach Implantation einer multifokalen IOL in den Kapselsack ist eine postoperative Refraktion nahe der Emmetropie entscheidend für ein gutes funktionelles Ergebnis. Venter et al. behandelten 80 pseudophake Augen, welche initial mit einer segmentalen bifokalen IOL versorgt wurden, mit der monofokalen asphärischen Sulcoflex 653L, um Brechungsfehler von −1,75 bis +3,25 dpt zu korrigierten. Ein Jahr nach Implantation der sulcusgestützten Linse lagen 93,8 % der Augen in einem Bereich von ±0,50 dpt [16]. Zur Presbyopiebehandlung stand bisher nur eine bifokale Version der Sulcoflex-Linse zur Verfügung. Huerva berichtete bei einer ehemals myopen Patientin über eine zufriedenstellende Behandlung mit einer bifokalen Sulcoflex 653F mit einer Brechkraft von +3,0 dpt und zusätzlicher Nahaddition von +3,5 dpt, da sie nach einer Kataraktoperation mit Implantation einer monofokalen IOL ein sphärisches Äquivalent von +2,25 dpt annahm [7].

### Das Duett-Verfahren

Es gibt einzelne Studien, welche die Implantationen einer additiven bifokalen IOL in einer Sitzung zusammen mit einer monofokalen Linse in den Kapselsack untersuchten. In einer prospektiven Studie von Cassagne et al. aus dem Jahr 2018 wurden 54 Augen von 27 Patienten in einer Sitzung mit einer monofokalen Kapselsacklinse und einer additiven diffraktiven bifokalen IOL (Reverso IOL, Cristalens, Lannion, Frankreich) mit einer +3,0-dpt-Addition behandelt. Ein Jahr nach der Operation zeigten sich gute Ergebnisse für den Nah- und Fernvisus (binokularer unkorrigierter Fernvisus = 0,10 ± 0,11 logMAR und binokularer unkorrigierter Nahvisus = 0,18 ± 0,12 logMAR) und eine hohe Patientenzufriedenheit von 98 % [2].

Liekfeld et al. verglichen an 52 Augen von 26 Patienten die bilaterale Implantation einer monofokalen IOL in den Kapselsack und gleichzeitig einer diffraktiven bifokalen sulcusgestützten IOL (Diff-sPB) mit einer konventionellen multifokalen IOL-Implantation (Diffractiva‑s) in den Kapselsack. Beide diffraktive Linsen waren von einem Hersteller (HumanOptics, Erlangen, Deutschland) und hatten eine Nahaddition von +3,5 dpt. Die Autoren kamen zu dem Schluss, dass die funktionellen Ergebnisse beider Verfahren gleichwertig waren und es keine signifikanten Unterschiede in der Patientenzufriedenheit und Lesegeschwindigkeit für jede Buchstabengröße zwischen den Gruppen gab. Im Vergleich zu der konventionellen Methode sei das kombinierte Verfahren jedoch bei Bedarf reversibel [11].

### Vorteil der Trifokalität gegenüber der Bifokalität

Sowohl klinische Studien als auch Untersuchungen an der optischen Bank zeigen, dass moderne diffraktive trifokale Optiken v. a. im Intermediärbereich den bifokalen überlegen sind, ohne jedoch zusätzliche oder vermehrte Nebenwirkungen zu induzieren [3, 5]. Da mit dem Duett-Verfahren in den letzten Jahren mit den bifokalen additiven IOL nur Bifokalität, mit trifokalen IOL im Kapselsack jedoch Trifokalität zu erzielen war, hat das Interesse am Duett-Verfahren in den letzten Jahren abgenommen. Mit der hier verwendeten neuen IOL lässt sich nun aber auch eine trifokale Optik in den Sulcus ciliaris einsetzen. Über das Duett-Verfahren unter Verwendung einer trifokalen additiven IOL gibt es derzeit noch keine Berichte in Peer-Review-Journals. Vergleichende Messungen der optischen Qualität zwischen der Kombination aus einer Sulcoflex trifocal und einer monofokalen RayOne Aspheric (Rayner Hove, UK) bestätigten eine ebenso gute optische Qualität hinsichtlich der Modulationsübertragungsfunktion im Fern‑, Intermediär- und Nahbereich wie bei einer klassischen diffraktiven trifokalen Kapselsack-IOL [9].

### Vorteile der Reversibilität

Einige Umstände können die Entfernung der multifokalen Optik notwendig werden lassen. Diese sind zum Teil präoperativ entweder nicht ersichtlich oder entwickeln sich erst im Laufe der Zeit.

Insgesamt treten photische Phänomene wie die Wahrnehmung von Lichtringen oder eine vermehrte Blendung bei multifokalen IOL bekanntermaßen häufiger auf als bei monofokalen Linsen [1]. Da eine IOL-Explantation aus dem Kapselsack mit einer vermehrten intraoperativen Komplikationsrate verbunden ist, jedoch in manchen Fällen die einzige Behandlungsoption darstellt, wäre die Möglichkeit einer einfachen Reversibilität eine große Erleichterung [4].

Die Verwendung von multifokalen Optiken bei myopen Patienten wird nach wie vor kontrovers diskutiert. Es wird vermutet, dass vorbestehende Netzhautveränderungen zu verminderten funktionellen Ergebnissen und einem erhöhten Auftreten von Nebenwirkungen führen können. Einzelne Studien konnten zwar zeigen, dass multifokale Optiken auch bei hoch myopen Patienten zu guten funktionellen Ergebnissen führen können, jedoch ist dies im Einzelfall schwer vorherzusagen [14]. Die Reversibilität ist insbesondere deswegen von Vorteil, weil trifokale Optiken oft bei jüngeren Patienten eingesetzt werden. Zwar kann die Implantation von trifokalen Linsen auch bei sehr jungen Patienten zu guten funktionellen Ergebnissen führen, zum Zeitpunkt der Implantation lässt sich aber nicht sicher vorhersagen, ob der Patient nicht später doch Erkrankungen entwickelt, die eine Kontraindikation für eine trifokale IOL wären [18]. Bei Patienten, die im Laufe des Lebens eine solche Pathologie am hinteren Augenabschnitt (z. B. eine AMD, ein Glaukom oder eine Amotio retinae) entwickeln, ist die aus dem Sulcus leicht zu entfernende trifokale Optik ein Vorteil.

Des Weiteren kann die Funktion der Netzhaut mit zunehmendem Alter abnehmen, was zu einer reduzierten Wirkung oder einer Unverträglichkeit der multifokalen Optik führen könnte. Auch in diesem Falle kann nur eine additive sulcusgestützte IOL im Gegensatz zu einer Kapselsack-IOL in einem relativ unkomplizierten Eingriff wieder entfernt werden.

Im Einzelfall ist es oftmals schwer vorherzusagen, bei welchen Patienten multifokale Optiken zu guten funktionellen Ergebnissen führen und in welchen Fällen Probleme auftreten. Deshalb bietet das Duett-Verfahren insbesondere mit den modernen trifokalen Optiken eine gute Erweiterung des Portfolios an Behandlungsoptionen für Patienten mit dem Wunsch nach Brillenunabhängigkeit.

Weitere Laboruntersuchungen und größere klinische Studien müssen zeigen, ob das Duett-Verfahren unter Verwendung moderner trifokaler Optiken vergleichbar gute Ergebnisse erzielt wie die trifokalen kapselsackfixierten Linsen.

## Fazit für die Praxis

- Das Duett-Verfahren mit neuen trifokalen IOL bietet eine Behandlungsmöglichkeit für Patienten mit dem Wunsch nach Brillenunabhängigkeit im Fern‑, Intermediär- und Nahbereich, wenn die Option einer unkomplizierten Reversibilität der Trifokalität gegeben sein soll.
- Im Falle einer funktionellen Verschlechterung, Visusreduktion oder dem Auftreten von Nebenwirkungen kann die Entfernung der additiven IOL vergleichsweise einfach erfolgen.

## Caption Electronic Supplementary Material


