# Erratum zu: Reversible Trifokalität durch das Duett-Verfahren

**DOI:** 10.1007/s00347-020-01109-2

**Published:** 2020-05-11

**Authors:** Ramin Khoramnia, Timur M. Yildirim, Hyeck-Soo Son, Grzegorz Łabuz, Christian S. Mayer, Gerd U. Auffarth

**Affiliations:** International Vision Correction Research Centre (IVCRC), Universitäts-Augenklinik Heidelberg, Im Neuenheimer Feld 400, 69120 Heidelberg, Deutschland

**Erratum zu:**

**Ophthalmologe 2020**

10.1007/s00347-020-01096-4

In der PDF-Version des Artikels war der Satz „Bisherige Linsenmodelle konnten dafür durch refraktive und/oder diffraktive Optiken nur 2 Foci (Fern- und Nahbereich) generieren.“ unvollständig. Die Online-Version wurde nachträglich korrigiert.

Wir bitten, das aktualisierte PDF zu beachten und den Fehler zu entschuldigen.

Die Redaktion

